# Prevalence of prediabetes, diabetes, diabetes awareness, treatment, and its socioeconomic inequality in west of Iran

**DOI:** 10.1038/s41598-022-22779-9

**Published:** 2022-10-25

**Authors:** Farhad Moradpour, Satar Rezaei, Bakhtiar Piroozi, Ghobad Moradi, Yousef Moradi, Negar Piri, Azad Shokri

**Affiliations:** 1 Social Determinants of Health Research Center, Research Institute for Health Development, Kurdistan University of Medical Sciences, Sanandaj, Iran; 2 Department of Public Health, School of Health, Kermanshah University of Medical Sciences, Kermanshah, Iran; 3 Department of Epidemiology and Biostatistics, Faculty of Medicine, Kurdistan University of Medical Sciences, Sanandaj, Iran; 4 Health Network of Dehgolan, Kurdistan University of Medical Sciences, Sanandaj, Iran

**Keywords:** Epidemiology, Endocrine system and metabolic diseases, Diseases, Health care, Molecular medicine, Risk factors

## Abstract

We aim to estimate the prevalence of prediabetes, and diabetes mellitus (DM). We estimated awareness, treatment, plasma glucose control, and associated factors in diabetes, as well as, socioeconomic-related inequality in the prevalence of diabetes and prediabetes. Data for adults aged 35–70 years were obtained from the baseline phase of the Dehgolan prospective cohort study (DehPCS). Diabetes status was determined as fasting plasma glucose (FPG) of ≥ 126 mg/dl and/or taking glucose lowering medication confirmed by a medical practitioner. Prediabetes was considered as 100 ≤ FPG ≤ 125 mg/dl. The relative concentration index (RCI) was used to exhibit socioeconomic inequality in the prevalence of prediabetes and DM. Prevalence of prediabetes and DM, diabetes awareness and treatment, and glycemic control of DM 18.22%, 10.00%, 78.50%, 68.91% and, 28.50%, respectively. Increasing age (p < 0.001), Increasing body mass index (BMI) (p < 0.05), ex-smoker (p < 0.01), family history of diabetes (FHD) (p < 0.001), and comorbidity (p < 0.001) were independent risk factors for DM. Age group of 46–60 (p < 0.05), ex-smoker (p < 0.05), FHD (p < 0.05) were increased chance of awareness. Current smokers (p < 0.05), and higher education increase the chance of glycemic control in DM. Both DM (RCI =  − 0.234) and prediabetes (RCI =  − 0.122) were concentrated significantly among less-educated participants. DM was concentrated significantly among poor (RCI =  − 0.094) people. A significant proportion of DM awareness and treatment can be due to the integration of diabetes into the primary health care system. The high prevalence of prediabetes and diabetes, which is affected by socioeconomic inequality and combined with low levels of glycemic control may place a greater burden on the health system. Therefore, awareness, receiving treatment, and glycemic control in people with diabetes, and the socioeconomic status of people have become increasingly important in the near future.

## Introduction

Today, diabetes mellitus (DM) is a worldwide public health challenge, imposing a significant burden on socioeconomic development and global public health^1,2^. International Diabetes Federation (IDF) has estimated that 537 million adults aged 20–79 are living with DM in 2021, and it is expected to rise to 783 million by 2045. In addition, 541 million adults with impaired glucose tolerance (IGT), are at higher risk for DM^3^.

The Prevalence of diabetes and prediabetes in people over 18 years was estimated at 14.15% and 24.79% respectively in 2021 in Iran. It represents a 45.5% increase in diabetes prevalence compared to 2016^4^. According to the last national survey of risk factors of non-communicable diseases (NCDs), 15.14% of the Iranian population over 25 years old had diabetes in 2021^4^. The number of people with diabetes in Iran is projected to increase to 9.2 million by 2030 if no effective prevention is incorporated^5^.

Due to the silent nature of diabetes, a significant portion of the affected population is unaware of their DM status^6^, and many people with diabetes are unaware of their complications due to uncontrolled blood glucose levels^7^. For example, according to national reports in 2021, the proportion of DM awareness in Iran was 73% and only 31% of people with diabetes in Iran have controlled hyperglycemia^4^.

Along with economic transition, urbanization, industrialization, and globalization, the rapid increase in diabetes prevalence have been occurred due to changes in socioeconomic status (SES), environmental, and lifestyle factors^8^. Environmental risk factors for DM are reasonably well established but the impact of SES in relation to DM is not well understood^9^.

Differences in health between socioeconomic groups are one of the major public health challenges worldwide^10^. Several literatures provide irrefutable evidence of SES as a determinant in prevalence and control of DM^8,10^. SES affects individual health behaviors through many aspects, including patient abilities, community or neighborhood support, health-related behaviors, access to care, and the care process^11^. This can lead to a lack of diabetes-related awareness and willingness to diagnose diabetes, which is important in glucose control in DM^12^. Undiagnosed diabetes can lead to a sudden adverse outcome, and if not diagnosed for a long time, it can progress into a serious problem in the patient's later life^12^.

Updating information on the prevalence, control, treatment, and awareness of diabetes and prediabetes is essential for prioritizing and planning health care services and achieving the goals of the World Health Organization (WHO) Action Plan for the Prevention and Control of NCDs in 2025^13^. In addition, measuring health inequalities is helpful in promoting equality and achieving Sustainable Development Goals (SDGs).

To our knowledge, previous studies have estimated the prevalence of diabetes in Iran, but limited studies have reported awareness, treatment, control of diabetes, and SES inequalities using clinical outcomes in addition to the self-reporting data. Therefore, this study aims to (1) describe the prevalence, control, treatment, and awareness of diabetes and prediabetes, (2) determine socioeconomic inequalities in the prevalence of diabetes and prediabetes using the Relative concentration index (RCI) among adults aged 35–70 years participating in the Dehgolan prospective cohort study (DehPCS).

## Methods

### Study population

The present study utilized enrollment phase data of the DehPCS. As a part of the prospective epidemiological research study in Iran (PERSIAN), DehPCS was conducted to investigate 35–70 years old permanent residents of Dehgolan. PERSIAN covers 18 cohort sits, including a representative sample of the major ethnicity all over Iran. Uniform questionnaires and data collection methods were used at all sites. PERSIAN aimed to follow up participants for the 15-year time period^14^.

**Figure 1:**
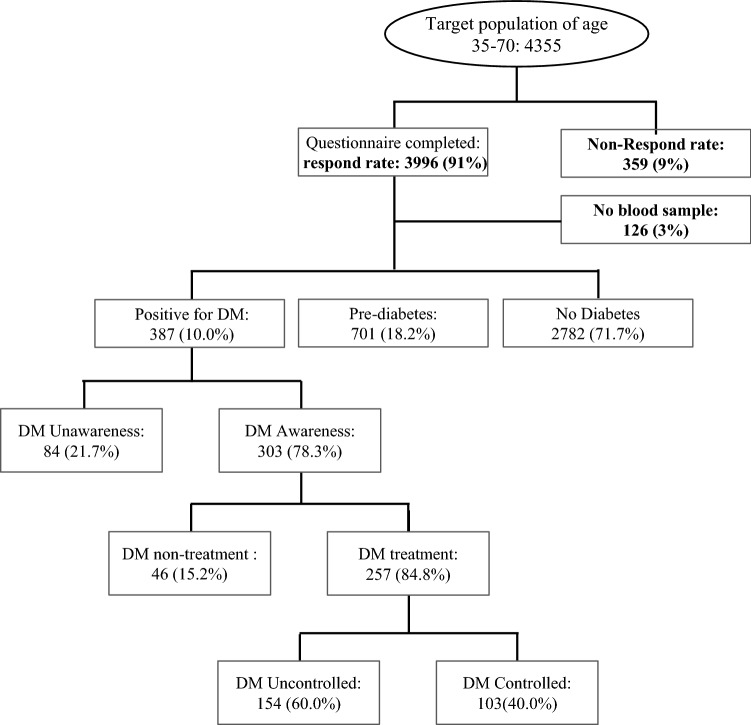
Flow diagram of participants in Dehgolan Prospective Cohort Study (DehPCS), 2018–2019, who respond to questionnaires and agreed for fasting blood glucose sampling.

Dehgolan County is a district of Kurdistan province, which is located in the west of Iran. Almost all 9000 residents 35–70 years old have a Kurdish ethnicity. By incorporating simple cluster sampling with a total number of 3996 participants who were enrolled in the study. The response rate of eligible people was 91%. The rationale and detail of the study have previously been published elsewhere^15^ (Fig. 1).

### Data collection and measurements

A local, trained personnel who spoke native Kurdish collected data in three dimensions including general, medical, and, nutritional information. Questionnaires consisted of demographic, socio-economic, medical/clinical/biological information, lifestyle, personal habit, anthropometry, medication use, and, nutritional status. All eligible participants are invited 1 week earlier to participate in the study. All participants were contacted by call reminder a day before the appointment schedule. All procedures performed in studies involving human participants were in accordance with the ethical standards of the institutional level and/or national level Medical Research Ethics Committee (MREC) and in line with the 1964 Helsinki declaration and its later amendments.

Upon arrival, the participants are been registered in the online software and received a unique code. In the first step, blood and urine samples were been taken from fasting participants. Height is been also measured at 0.1 cm accuracy with Seca stadiometer. To measure weight, we used Seca scale. Body mass index (BMI) was calculated by dividing weight (kg) by height (cm). Subsequently, based on world health organization guidelines^16^ BMI was categorized into three groups, normal (≤ 24.9), overweight (≤ 29.9) and, obese (≥ 30.0). Education was categorized as follows: illiterate (zero years of education), 1–6 years of education, 7–12 years of education, and university (more than 12 years of education).

Economic status was defined based on the Wealth index. Wealth index was calculated according to the selected living assets, including having a freezer, washing machine, dishwasher, vacuum cleaner, color TV (no color TV or regular color TV vs. Plasma color Tv), access to internet, motorcycle, car (no access, access to a car with price of < 50 million Tomans, and access to a car with price of > 50 million Tomans), owning a mobile, owning a PC or laptop, international trips in their lifetime. The total wealth score was calculated based on the multiple correspondence analysis (MCA) of assets data^17^. Then, participants were ranked and divided into three categories of wealth index based on 2 quantiles (cut point), by dividing the range of probability distribution into intervals with an equal probability. The groups were named poor, middle, and rich respectively.

Individuals who smoked more than 100 cigarettes during their lifetime are considered as smokers. However, if someone had smoked during the last month was grouped as current, and if they had not smoked as an ex-smoker. Illicit drug use was defined as using illicit drugs once per week for at least 6 months. Alcohol consumption was defined as drinking approximately 200 ml of beer or 45 ml of liquor, once per week for at least six months. Participants physical activity was measured by self-report of time spent on recreation, work, sport, and other activity during 1 week. The mean 24-h metabolic equivalent task (MET) score was calculated based on the weight, activity type, and time spent on activities. Then, METs were divided into following categories: 24–36.5 (low), 36.6–44.9 (moderate), and ≥ 45 (vigorous)^18^. Lipid profile was examined after 9–12 h of fasting. Triglyceride (TG), HDL, and LDL-cholesterol were measured through colorimetric and total cholesterol by enzymatic method. Dyslipidemia was determined based on the total cholesterol ≥ 240 (mg/dl), and/ or TG ≥ 200 (mg/dl) and/or LDL ≥ 160 (mg/dl), and/or HDL < 40 (mg/dl), and/or use of medication to treat these conditions^19^.

Hypertension was measured by a hand-held aneroid sphygmomanometer. After 15 min of rest in a sitting position, blood pressure (BP) was measured twice in each arm with 30 min of time intervals. The average of four systolic and diastolic (SBP & DBP) values were used to determine BP. Those who had SBP ≥ 140 and/or DBP ≥ 90 and/ or use anti-hypertensive medication were considered as hypertensive individuals^20^.

Diabetes status was determined as abnormal fasting plasma glucose (FPG) and/or current history of taking glucose lowering medication, and/or if was confirmed by a medical practitioner. Prediabetes and diabetes were considered as 100 ≤ FPG ≤ 125 mg/dl and FPG ≥ 126 mg/dl respectively^21^. Awareness is determined by individual self-reported diabetes among people with diabetes. Patients who received medication and his/her FPG less than 126 mg/dl were considered as controlled FPG.

### Statistical analysis

Prevalence of prediabetes, and DM were defined as the number of affected participants divided by the number of all participants. The proportion of the awareness, treatment, and FPG control were also estimated by the available standard method. A 95% confidence interval (CI) were calculated for all estimation. The adjusted logistic regression method was used to investigate the independent relationship of all variables with a p-value less than 0.3 in the univariable analysis. Crude and adjusted odds ratio (OR) with their CI were provided with a significant level of 0.05. RCI and concentration curve (CC) was used to examine the SES-related inequality by years of education and wealth index in the prevalence of prediabetes and diabetes in the study population^22^. Data were analyzed using STATA software version 16.0 (Stata Corp, College Station, TX, USA).

### Ethics approval and consent to participate

The ethical review board at Kurdistan University of Medical Sciences has approved study design and protocol of this study under the code of IR.MUK.REC.1400.149. For experiments involving human participants, informed consent have been obtained.

## Results

In total 3996 participants enrolled in the study. 43.74% of participants were male, with a mean age of 48.72 ± 8.91 years. generally, 91.87% of participants were married and, 31.21% were illiterate. 32.3% of participants had BMI ≥ 30 and may have obesity complications. 15.19% were current smokers, 11.34% used illicit drugs, and more than 12% of them were alcohol users. In total, 31.55% of participants reported a family history of diabetes, and 60.11% of them suffered from at least one comorbidity including dyslipidemia (38.61%), hypertension (8.01%), or both (13.49%). The prevalence of dyslipidemia and hypertension among participants were 52.10% and 21.5% respectively (Table 1).

**Table 1:** Prevalence of pre-diabetes, diabetes, awareness, treatment, and FBS control across different groups in DehPCS.

	Pre-diabetes% (CI)	Diabetes% (CI)	Awareness% (CI)*	Treatment% (CI)*	Control% (CI)*
Overall (3996)	18.22 (16.99–19.52)	10.00 (9.09–10.97)	78.50 (74.10–82.32)	84.82 (80.30–88.45)	40.00 (34.73–45.93)
**Gender**					
Male (1748)	15.80 (14.80–17.69)	8.68 (7.43–10.12)	72.11 (64.30–78.77)	87.73 (79.98–92.76)	36.73 (27.76–46.73)
Female (2248)	20.13 (18.43–21.94)	11.02 (9.78–12.41)	82.43 (77.05–86.76)	83.25 (77.34–87.85)	41.81 (34.74–49.23)
**Age groups**					
35–45 (1786)	14.51 (12.90–16.28)	4.46 (3.58–5.54)	66.23 (55.60–76.87)	80.39 (67.17–89.14)	43.75 (30.49–57.97)
46–60 (1724)	21.14 (19.15–23.27)	12.34 (10.85–14.01)	82.67 (77.44–87.91)	81.76 (75.20–86.89)	40.14 (32.50–48.29)
> 60 (486)	22.84 (18.96–27.25)	21.94 (18.44–25.89)	78.64 (70.68–86.60)	93.90 (86.13–97.44)	37.50 (27.60–48.59)
**Marital status**					
Married (3671)	17.71 (16.45 19.05	9.63 (8.70–10.64)	78.59 (73.90–82.63)	84.70 (79.86–88.55)	40.16 (34.17–46.47)
Single (325)	24.20 (19.55 29.55)	14.20 (10.77–18.49)	77.78 (63.36–87.63)	85.71 (69.87–93.95)	38.71 (23.41–56.62)
**Education years**					
Illiterate (1247)	22.83 (20.40–25.46)	15.62 (13.68–17.78)	84.57 (79.39–89.75)	83.65 (77.3–88.64)	35.25 (27.74–43.57)
1–5 (1112)	17.73 (15.49–20.22)	9.29 (7.70–11.18)	76.00 (67.58–83.38)	82.89 (72.68–89.82)	43.93 (32.48–56.08)
6–12 (1123)	15.62 (13.54–17.96)	6.17 (4.88–7.77)	65.67 (54.21–77.13)	90.91 (78.08–96.55)	45.83 (32.36–59.94)
University (514)	14.68 (11.77–18.14)	6.22 (4.41–8.72)	77.42 (59.57–88.86)	87.50 (67.51–95.93)	45.45 (26.40–65.94)
**Economic status**					
Lower (1345)	17.92 (16.05–19.96)	11.27 (9.76–12.98)	78.44 (71.54–84.05)	84.73 (77.48–89.95)	41.74 (33.05–50.97)
Middle (1305)	19.03 (16.84–21.44)	10.89 (9.20–12.86)	75.21 (66.72–82.11)	83.52 (74.40–89.83)	36.14 (26.52–47.02)
Upper (1320)	17.72 (15.70–19.94)	7.78 (6.42–9.40)	82.47 (73.57–88.83)	86.25 (76.80–92.24)	42.11 (31.52–53.47)
**BMI**					
Normal weight (989)	9.59 (7.84–11.69)	5.99 (4.65–7.69)	80.70 (68.37–89.00)	86.96 (74.00–94.04)	35.00 (21.09–50.08)
Over-weight (1705)	17.95 (16.09–19.97)	11.66 (10.20–13.30)	81.25 (75.10–86.17)	84.61 (78.04–89.48)	41.55 (33.70–49.85)
Obese (1286)	25.17 (22.75–27.76)	10.85 (9.24–12.69)	74.26 (66.24 -80.93)	84.16 (75.65–90.08)	39.13 (29.68–49.47)
**Cigarette smoking**					
No smoker (3024)	19.12 (17.69–20.64)	9.28 (8.28–10.39)	78.31 (73.00–82.82)	85.45 (80.02–89.59)	38.22 (31.57–45.34)
Ex-smoker (326)	18.41 (14.27–23.42)	18.04 (14.18–22.67)	89.47 (78.44–95.20)	84.31 (71.59–91.98)	42.55 (29.30–56.97)
Current smoker (600)	13.43 (10.80–16.59)	9.64 (7.49–12.32)	67.86 (54.60–78.70)	81.58 (66.03–90.98)	45.71 (30.16–62.15)
**Drug use**					
No (3502)	18.50 (17.18–19.90)	10.14 (9.17–11.21)	78.78 (74.12–82.79)	83.76 (78.86–87.71)	41.32 (35.26–47.66)
Yes (448)	15.88 (12.63–19.78)	9.38 (6.98–12.50)	75.61 (60.26–86.37)	93.55 (77.48–98.39)	29.03 (15.80 -47.13)
**Alcohol use**					
No (3470)	18.54 (17.21–19.95)	10.49 (9.50–11.57)	79.04 (74.46–82.98)	83.51 (78.66–87.43)	40.96 (34.50 -47.21)
Yes (481)	15.98 (12.84–19.72)	6.90 (4.92–9.59)	71.87 (54.12–84.70)	1.00	29.17 (14.53–49.92)
**Family history of Diabetes**					
No (2436)	17.01 (15.50–18.64)	7.64 (6.63–8.78)	70.00 (62.89–76.26)	86.51 (79.33–91.46)	41.46 (33.07–50.38)
Second degree (430)	16.07 (12.76–20.05)	9.62 (7.13–12.85)	95.00 (82.02–98.75)	81.58 (66.03–90.98)	53.33 (35.73–70.14)
First degree (823)	21.06 (18.23–24.20)	14.18 (11.93–16.77)	80.70 (72.39–86.96)	83.69 (74.65–89.94)	35.80 (26.11–46.81)
First and second degree (283)	25.64 (20.45–31.62)	18.98 (14.76–24.06)	90.38 (78.84 -95.95)	85.11 (71.84–92.75)	32.50 (19.85–48.34)
**MET-hour (daily)**					
Low (1619)	18.66 (16.73–20.76)	11.54 (10.05–13.22)	80.00 (73.49–75.23)	88.19 (81.80–92.55)	41.48 (33.45–49.99)
Moderate (1787)	18.51 (16.69–20.48)	9.63 (8.32–11.11)	78.44 (71.54–84.04)	83.97 (76.62–89.32)	40.68 (32.16–49.79)
Vigorous (553)	15.94 (12.99–19.41)	6.92 (5.05–9.40)	70.27 (53.82–82.74)	73.08 (53.21–86.63)	28.57 (13.39–50.87)
**Comorbidity**					
No (1594)	13.43 (11.82–15.22)	3.89 (3.05–4.96)	70.97 (58.51–80.91)	88.64 (75.39–95.21)	42.22 (28.74–56.97)
Dyslipidemia (1543)	19.52 (17.47–21.74)	11.63 (10.08–13.38)	76.92 (69.95–82.68)	82.31 (74.74–87.97)	38.74 (30.11–48.13)
Hypertension (320)	21.36 (17.05–26.40)	10.94 (7.96–14.86)	82.86 (66.68–92.11)	79.31 (60.86–90.43)	50.00 (31.60–68.40)
Both (539)	29.85 (25.63–34.45)	24.01 (20.48–27.93)	83.33 (75.54–89.00)	88.00 (80.00–93.07)	37.63 (28.36–47.91)

*Each proportion were calculated based on the previous column.

### Prevalence, awareness, treatment, FPG control

Of all 3996 participants 18.22 (CI 16.99–19.52), and 10.00 (CI 9.09–10.97) were pre diabetic and people with diabetes respectively. The mean and standard deviation of FPG for all samples, females, and males, were 98.31 ± 32.53, 99.26 ± 34.02, and 97.09 ± 30.48 respectively. The proportion of diabetes significantly increased with age in both males and females (p-value for trend < 0.001). The proportion of prediabetes, diabetes, awareness, and FPG control were higher in females than males but, the probability of receiving treatment was lower for females. Diabetes decreased with education years for both males and females (p-value for trend 0.01 and < 0.001 respectively). Women in higher economic classes experienced a lower prevalence of diabetes (p-value for trend = 0.011).

Both diabetes and prediabetes prevalence increased linearly based on the groups formed by the closeness of family relationship (p-value for trend < 0.001). However, the proportion of patients with controlled glycemic level was lower in participants whose familial relationships with people with diabetes were stronger.

Decreasing trend (p-value = 0.009) for diabetes prevalence was seen across the intensity of physical activity but, a sharp increasing trend was seen based on groups formed by comorbidity (p-value for trend < 0.001).

Among all 387 people with diabetes, 303 (78.50%) aware of their condition, 266 (68.73%) take glycemic lowering medication, and 110 (28.72%) were controlled their FPG level (Table 1).

### Prediabetes and diabetes associated factors

Although, the crude odds of prediabetes and diabetes were greater in females (OR 1.35, p < 0.001 and OR 1.30, p = 0.016 respectively), adjusted odds were equally distributed among both males and females (Table 2). Age was directly associated with both prediabetes and diabetes (p < 0.001) and education was reversely related to the diabetic and prediabetic patients. Divorced/widowed/single patients were more likely to get diabetes in the crude model.

**Table 2:** Correlates of prediabetes, diabetes, awareness, treatment and FBS control according to the logistic regression in DehPCS.

	Prediabetes		Diabetes		Awareness		Treatment		Control	
	Crud OR (CI)	Adjusted OR (CI)	Crud OR (CI)	Adjusted OR (CI)	Crud OR (CI)	Adjusted OR (CI)	Crud OR (CI)	Adjusted OR (CI)	Crud OR (CI)	Adjusted OR (CI)
**Gender**										
Female	1	1	1	1	1	1	1	1	1	1
Male	0.74 (0.63–0.89)^c^	1.13 (0.86–1.49)	0.77 (0.62–0.95)^a^	0.91 (0.64–1.31)	0.55 (0.34–0.90)^a^	0.58 (0.26–1.28)	1.44 (0.72–2.87)	1.34 (0.43–4.15)	0.81 (0.49–1.34)	0.32 (0.14–0.75)^b^
**Age groups**										
35–45	1	1	1	1	1	1	1	1	1	1
46–60	1.58 (1.31–1.90)^c^	1.50 (1.20–1.87)^c^	3.02 (2.30–3.96)^c^	2.59 (1.88–3.57)^c^	2.48 (1.36–4.49)^b^	2.55 (1.19–5.48)^a^	1.09 (0.49–2.42)	1.59 (0.61–4.16)	0.86 (0.45–1.66)	1.28 (0.56–2.93)
> 60	1.74 (1.33–2.29)^c^	1.66 (1.16–2.38)^b^	6.02 (4.39–8.26)^c^	4.68 (3.03–7.24)^c^	1.90 (.97–3.70)	1.89 (0.72–4.94)	3.76 (1.20–11.73)^a^	6.08 (1.52–24.32)^b^	0.77 (0.37–1.60)	1.51 (0.56–4.07)
**Marital status**										
Married	1	1	1	1	1	–	1	–	1	–
Divorced/widow/single	1.48 (1.11–1.98)^b^	1.30 (0.94–1.79)	1.55 (1.11–2.17)^b^	0.92 (0.62–1.35)	0.95 (0.45–2.02)	–	1.08 (0.40–2.96)	–	0.94 (0.44–2.02)	–
**Education years**										
Illiterate	1	1	1	1	1	1	1	1	1	1
1–5	0.73 (0.59–0.90)^b^	0.94 (0.73–1.21)	0.55 (0.43–0.72)^c^	0.92 (0.67–1.26)	0.58 (0.32–1.06)	0.66 (0.29–1.46)	0.95 (0.46–1.97)	1.79 (0.66–4.87)	1.44 (0.79–2.62)	2.44 (1.13–5.28)^a^
6–12	0.63 (0.50–0.78)^c^	0.89 (0.66–1.20)	0.36 (0.27–0.48)^c^	0.80 (0.53–1.21)	0.35 (0.18–0.66)^c^	0.39 (0.15–1.04)	1.95 (0.64–5.93)	2.96 (0.75–11.69)	1.55 (0.80–3.02)	4.02 (1.45–10.45)^b^
University	0.58 (0.43–0.78)^c^	0.89 (0.59–1.27)	0.36 (0.24–0.53)^c^	0.87 (0.50–1.55)	0.63 (0.25–1.58)	0.49 (0.12–1.95)	1.36 (0.38–4.92)	2.61 (0.44–14.94)	1.53 (0.62–3.79)	4.79 (1.26–18.18)^a^
**Economic status**										
Lower	1	–	1	1	1	1	1	–	1	–
Middle	1.07 (0.88–1.31)	–	096 (0.75–1.23)	1.04 (0.79–1.36)	0.83 (0.48–1.45)	0.98 (0.58–1.87)	0.91 (0.44–1.89)	–	0.79 (0.44–1.41)	–
Upper	0.99 (0.81–1.20)	–	0.66 (0.52–0.86)^b^	0.87 (0.63–1.20)	1.29 (0.68–2.45)	1.73 (0.78–3.83)	1.13 (0.51–2.50)	–	1.02 (0.56–1.82)	–
**BMI**										
Normal weight	1	1	1	1	1	1	1	–	1	1
Over-weight	2.06 (1.59–2.67)^c^	1.95 (1.49–2.56)^c^	2.07 (1.52–2.81)^c^	1.99 (1.42–2.79)^c^	1.04 (0.49–2.19)	0.79 (0.32–1.93)	0.82 (0.31–2.16)	–	1.32 (0.63–2.74)	1.80 (0.78–4.13)
Obese	3.17 (2.45–4.10)^c^	2.79 (2.11–3.69)^c^	1.91 (1.38–2.63)^c^	1.56 (1.09–2.25)^a^	0.69 (0.32–1.48)	0.40 (0.15–1.03)	0.80 (0.29–2.19)	–	1.19 (0.55–2.58)	1.57 (0.64–3.86)
**Cigarette smoking**										
No smoker	1	1	1	1	1	1	1	1	1	1
Ex-smoker	0.95 (0.69- 1.31)	0.82 (0.58–1.16)	2.15 (1.57–2.94)^c^	1.64 (1.13–2.36)^b^	2.35 (0.96–5.75)	2.91 (1.08–7.86)^a^	0.91 (0.39–2.13)	0.58 (0.21–1.59)	1.19 (0.63–2.28)	1.84 (0.88–3.86)
Current smoker	0.66 (0.50–0.86)^c^	0.69 (0.50–0.94)^a^	1.04 (0.77–1.41)	1.28 (0.88–1.86)	0.58 (0.31–1.10)	0.69 (0.31–1.53)	0.75 (0.31–1.86)	0.48 (0.17–1.39)	1.36 (1.66–2.81)	2.63 (1.06–6.52)^a^
**Drug use**										
No	1	–	1	–	1	–	1	–	1	1
Yes	0.83 (0.63–1.10)	–	0.92 (0.65–1.29)	–	0.58 (0.26–1.31)	–	2.81 (0.65–12.20)	–	0.58 (0.26–1.31)	0.58 (0.23–1.45)
**Alcohol use**										
No	1	–	1	1	1	–	1	–	1	–
Yes	0.84 (0.34–1.10)	–	0.63 (0.43–0.92)^a^	0.65 (0.42–0.99)^a^	0.68 (0.30–1.53)	–	1.00	–	0.59 (0.24–1.48)	–
**Family history of diabetes**										
No	1	1	1	1	1	1	1	–	1	1
Second degree	0.93 (0.70–1.25)	1.02 (0.75–1.38)	1.29 (0.90- 1.84)	1.74 (1.18–2.56)^b^	8.14 (1.89–34.96)^b^	9.43 (2.05–43.34)^b^	0.69 (0.26–1.81)	–	1.61 (0.72–3.59)	2.27 (0.93–5.57)
First degree	1.30 (1.05–1.61)^a^	1.32 (1.06–1.35)^a^	1.10 (1.55–2.56)^c^	2.43 (1.85–3.18)^c^	1.79 (1.02–3.15)^a^	2.16 (1.12–4.01)^a^	0.80 (0.38–1.70)	–	0.79 (0.44–1.40	0.87 (0.46–1.62)
First and second degree	1.68 (1.23–2.30)^c^	1.77 (1.28–2.46)^c^	2.83 (2.02–3.97)^c^	3.61 (2.48–5.24)^c^	4.03 (1.52–10.68)^b^	6.88 (2.22–21.28)^b^	0.89 (0.34–2.31)	–	0.68 (0.32–1.44)	0.65 (0.28–1.49)
**MET-hour (daily)**										
Low	1	–	1	1	1	–	1	1	1	–
Moderate	0.99 (0.82–1.19)	–	0.82 (0.65–1.02)	1.01 (0.78–1.29)	0.91 (0.54–1.53)	–	0.70 (0.35–1.39)	0.96 (0.46–2.00)	0.97 (0.58–1.60)	–
Vigorous	0.83 (0.63–1.08)	–	0.57 (0.39–0.82)^b^	0.89 (0.59–1.36)	0.59 (0.27–1.31)	–	0.36 (0.13–0.99)^a^	0.24 (0.07–0.82)^a^	0.56 (0.21–1.54)	–
**Comorbidity**										
No	1	1	1	1	1	1	1	–	1	–
Hypertension	1.56 (1.28–1.91)^c^	1.46 (1.18–1.80)^c^	3.25 (2.41–4.39)^c^	2.92 (2.14–3.98)^c^	1.36 (0.71–2.62)	1.42 (0.67–3.00)	0.60 (0.21–1.68)	–	0.86 (0.43–1.75)	–
Dyslipidemia	1.75 (1.28–2.40)^c^	1.41 (1.01–1.97)^a^	3.03 (1.97–4.68)^c^	1.83 (1.15–2.89)^b^	1.98 (0.70–5.57)	2.27 (0.65–7.97)	0.49 (0.14–1.79)	–	1.37 (0.52–3.61)	–
Both	2.74 (2.12–3.55)^c^	1.98 (1.49–2.63)^c^	7.80 (5.63–10.81)^c^	4.46 (3.13–6.37)^c^	2.04 (0.99–4.29)	1.72 (0.73–6.06)	0.94 (0.31–2.85)	–	0.82 (0.40–1.71)	–

^a^p-value < 0.05, ^b^p-value < 0.01, ^c^p-value < 0.001.

Results of the adjusted model showed BMI increased odds of diabetes and prediabetes independently of other variables (p < 0.001). Current smoking was reversely associated with prediabetes (p < 0.05), and ex-smoking directly increases the risk of diabetes (p < 0.01) independently of other variables. History of alcohol use had moderately decreased odds of diabetes (p < 0.05). Both pre-diabetes and diabetes independently had a significant dose–response relationship with those who have reported positive family history of diabetes (Table 2). Comorbidity with hypertension and dyslipidemia were increased risk of diabetes (p < 0.001) and pre-diabetes (p < 0.01) in both univariable and multivariable analysis (Table 2).

### DM awareness associated factors

Females have more chance to become aware of their diabetes condition (crude OR 1.82, p < 0.001). Age was directly associated with awareness (crude OR 1.03, p = 0.023). A lower chance of awareness was observed in participants with higher education years (crude OR 0.94, p = 0.011). Positive history of diabetes in the family especially in their second-degree family members increased awareness of diabetes condition (p < 0.001).

Multivariable regression results showed that the awareness was higher in the older ages independently of other variables, as the age group of 46–60 showed the strongest relationship with awareness (Adjusted OR 2.55, 95% CI 1.19–5.48). Previously smokers were more likely to be aware of their DM condition than non-smokers were (Adjusted OR 2.91, 95% CI 1.08–7.86). Participants with a positive history of diabetes in their families were more probable to be aware of their DM situation. Participant’s comorbid with hypertension and dyslipidemia were more aware of their diabetes condition, but it was not statistically significant (Table 2).

### DM treatment associated factors

Males and females have an equal chance to get DM treatment (p = 0.301). In addition, more older participants have a higher probability of receiving treatment (p = 0.026), and participants with a higher level of physical activity were more likely to get treatment. (p = 0.043).

After adjusting for the effect of covariates in the multivariable regression model, participants more than 60-years-old were about 6 times more likely to receive treatment than those in 35–45 (Adjusted OR 6.08, 95% CI 1.52–24.32). Also, physical activity MET score ≥ 45 (vigorous) significantly decreased the 76% probability of getting DM treatment independently of other variables (Adjusted OR 0.24, 95% CI 0.07–0.82). Being male and of a higher education level increased the chance of receiving treatment, while smoking reduced the likelihood of receiving treatment. However, these relationships were not statistically significant (p > 0.05) (Table 2).

### DM control associated factors

In the univariate analysis, the control of glycemic level was equally distributed among different levels of independent variables. However, in the multivariable logistic regression, after adjusting for different covariates, glycemic control was significantly different for gender, education, and smoking status. The odd of diabetes control was 68% lower in male than female participants (OR 0.32, 95% CI 0.14–0.75). There was a significant dose–response relationship between education levels and glycemic control in diabetes patients. It was about 2.4, 4.0, and 4.8 times more than illiterate for primary, high school, and university degrees respectively (OR 2.44, 4.02 and, 4.78 respectively). Ex-smokers and current smokers were more probable to control their glycemic index than non-smokers. It was only statistically significant in current smokers with odds of about 2.5 times higher than non-smokers (OR 2.63, 95 CI 1.06–6.52) (Table 2).

### SES-related inequality

**Figure 2:**
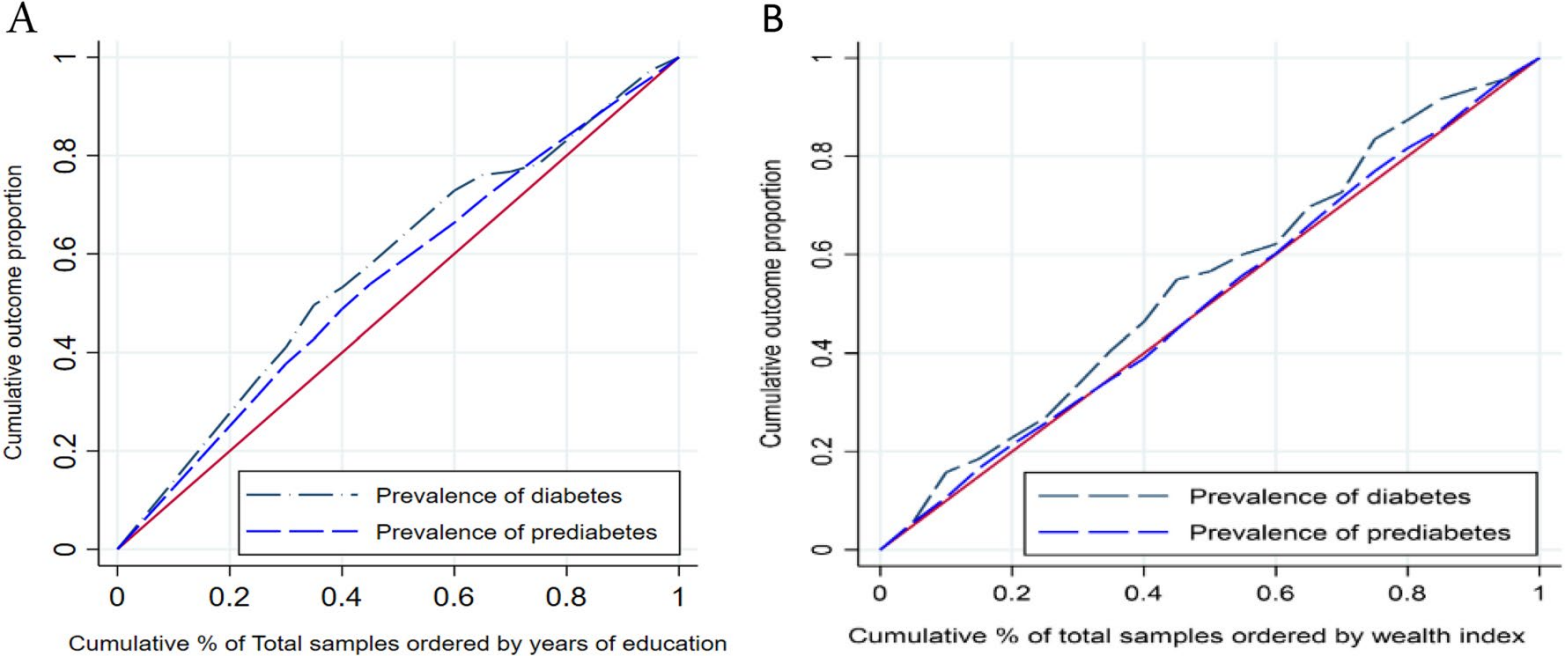
Concentration index for prediabetes and diabetes according to the education years (**A**) and wealth index (**B**).

The results of socioeconomic-related inequality for the prevalence of prediabetes and diabetes are reported in Table 3. As indicated, diabetes was concentrated among the poorer people (RCI =  − 0.094, 95% CI − 0.155 to − 0.033) and people with lower education levels (RCI =  − 0.234, 95% CI − 0.293 to − 0.175). We also observed predicates are more prevalent among the poor and among the less-educated individuals (RCI =  − 0.122, 95% CI =  − 0.170 to − 0.070). However, the results for the prevalence of prediabetes by wealth index are not significant (p-value = 0.509). For prediabetes and diabetes prevalence, education-related inequality was higher than wealth-related inequality (Fig. 2). As indicated in Fig. 2, the CC for the prevalence of diabetes and prediabetes lies on the perfect line; meaning that the higher prevalence of diabetes and prediabetes are more concentrated among the socioeconomically disadvantaged population (less educated and lower income).

**Table 3:** Socioeconomic-related inequality for prevalence of prediabetes and diabetes.

	Relative concentration index	Confidence interval 95%	p-value
**Wealth index**			
Prediabetes	− 0.016	− 0.065 to 0.032	0.509
Diabetes	− 0.094	− 0.155 to − 0.033	0.002
**Education status**			
Prediabetes	− 0.122	− 0.170 to − 0.070	< 0.001
Diabetes	− 0.234	− 0.293 to − 0.175	< 0.001

## Discussion

The prevalence of DM and prediabetes among 3996 participants was 10% and 18%, respectively. About 78% of patients were aware of their disease status and 84.82% were under treatment. However, glycemic control was observed in only 40% of the treatment on people with diabetes. According to the results, the DM prevalence in this study was less than the national estimate of 15.0% in Iran in 2021^13^. Similar studies with the same age groups, in Shahroud (12%)^23^, Ahvaz (15%)^24^, and Yazd (24%)^25^ reported a higher proportion, and Ravansar in Kermanshah (8.19%)^6^ and Valashahr in Fars (9.19%)^26^ reported less prevalence of diabetes. Also, this prevalence was still fewer than that of Afghanistan^27^, and Pakistan^28^ and higher than that of Bangladesh^29^ and Nepal^30^. This may be due to differences in people's age distribution, demographic characteristics, and lifestyles.

The association between smoking and DM or prediabetes was challenging in the current study. Although much evidence has shown an association between smoking, DM, and prediabetes^31–33^, in the present study, after eliminating confounding factors, smoking had only an effect on prediabetes and no significant effect on DM. In the present study, ex-smokers had a higher risk of developing DM compared to non-smokers. In a meta-analysis of prospective studies, the risk of DM after quitting smoking was also observed to steadily decrease and achieve a level of risk compared to those who never smoked^33^. Conversely, some studies similar to the current study showed quitting smoking might increase the risk of DM^33,34^. Probably due to weight gain and increased waist circumference after smoking cessation, the risk of DM increased^33^. There was a protective association between alcohol consumption and a chance of developing DM in this study. Other studies showed some inconsistencies in alcohol consumption and its effect on DM. In the meta-analysis study, low and moderate consumption reduced DM by 28%. In addition, excessive alcohol consumption had no effect on DM incidence. Low and moderate alcohol consumption could improve insulin sensitivity and reduce fasting insulin concentrations and glycosylated hemoglobin^35^. In contrast, in a cohort study on Korean adults, alcohol consumption ≥ 2 units per day (≥ 16 g/day) significantly increased the risk of DM^36^, so high alcohol consumption may be considered a risk factor for DM. In this regard, probably due to legal and religious restrictions on alcohol consumption in Iran, consumers are likely to be of low or moderate types. According to the results of previous studies, the diabetes family history is considered as an independent factor of DM and prediabetes. Also, the risk of DM was much higher in people who suffered from hypertension or dyslipidemia^37,38^. This has also been seen in previous studies.

DM awareness in the present study was 78%, which was almost the same as the national estimate (awareness of 79.6%). It is different in other parts of Iran. For example, DM awareness in Yazd and Kerman was about 97–95%^25^ while in Ahvaz, it was 40%^24^ and in the neighboring province (Kermanshah), it was similarly 75%^6^. In other countries such as China^39^, the US^40^, and Malaysia^41^, it was reported 52.5%, 71.5%, and 65.2%, respectively. Over the past years in Iran, the prevalence of undiagnosed diabetes has decreased from 45.7 to 24.7%^42^. The results of the present study paradoxically showed DM awareness had an inverse association with higher income levels while in some studies; individuals in higher income families had more awareness of diabetes^43–45^. Other studies in Iran and other countries did not report a significant association between diabetes and awareness^44–46^. Higher-income people may be able to receive health education and services more easily, but in recent years, with the expansion of screening programs of the health system to identify people with diabetes at the primary levels, and by reducing financial barriers, income does not have such an impact on access to primary health services. Similarly, in the present study, the diabetes family history^6,47^, age^6,48^ and smoking cessation^6^ had a direct association with DM awareness. In fact, people with a family history of diabetes show more health protective behaviors, especially weight control behaviors, than those without a family history of diabetes. In addition, with aging due to increased experiences and risk of diabetes, it seems that increasing awareness is logical. Ex-smokers are also more likely to develop DM than non-smokers^39^, so people may also develop other positive health-related behaviors at the same time as quitting smoking.

In this study, approximately 85% of people with diabetes were under treatment, which was higher than the statistic of 33% in Iran^49^ in previous years and the rate reported in neighboring provinces (75%)^6^. Treatment was reported in Malaysia 87%^41^, in Portugal 80%^50^, in the UA 71%^40^ and in villages of China 62%^51^. Therefore, receiving treatment among people with diabetes seems desirable compared to other studies. Also, based on the results of this research and other studies, treatment increases among age groups of 60 years and older^51^.

Despite the fact that a significant proportion of patients were treated in this study, only 40% of them controlled their blood sugar, which was almost similar to the national estimate (41.2%)^13^, and was lower than the rates reported for Portugal (63%)^50^, and the United States (50%)^40^ while it was higher than those for Kermanshah (33%)^52^, the national study in Iran (33%)^52^, Malaysia (22%)^41^, and villages of China (22%). In the results of previous studies, the low the proportion of glucose control has been stated as an indicator of inappropriate quality of services provided to people with diabetes^52^. Women also controlled at diabetes relatively higher rates than men. Studies also showed women were more adherent than men^53^. In the present study, the chance of diabetes control among ex-smoker was higher than among smokers and non-smokers. However, in other studies, smoking cessation has been associated with significant weight gain and body weight gain after quitting smoking may worsen glycemic control^54–56^. The results of our study are inconsistent with this finding perhaps because people who wish to quit smoking at the same time follow other health-related recommendations and people with diabetes who quit smoking also pay more attention to their blood sugar control and adherence to treatment^33^.

Finally, inequality analysis showed the prevalence of pre-diabetes and diabetes was significantly more concentrated among people with lower education, and financial levels. Other studies also showed the effect of educational inequality on diabetes^1^ and in a study, the likelihood of developing diabetes and pre-diabetes among people with low education was twice as high as those with moderate and high education levels whereas it clearly decreased in the higher education group^57^. Higher education generally seems to lead to better working conditions, higher incomes and better housing, and thus affects a person’s ability to take a more favorable social position. In other words, people with lower education may be exposed to risk factors and susceptible to T2 diabetes compared to their counterparts due to more unfavorable living conditions^1,58^. Similarly, in other studies, a positive association was observed between diabetes, pre-diabetes, and wealth status^59–61^. Even in a study, it has been noted that undesirable SES early in life are associated with an increased risk of developing pre-diabetes and diabetes in adulthood^62^.

In terms of socioeconomic inequality, our study indicated that diabetes and prediabetes are more prevalent among less educated people and among people with lower wealth scores. These findings are consistence with the studies conducted in other countries^11,63^. A study conducted by Al-Hanawi et al. in Saudi Arabia indicated that diabetes prevalence was concentrated among the poor and among people with less education. They also concluded that education-related inequality was higher than income-related inequality^64^.

It is worth noting that 97% of the participants underwent blood sampling, which was much higher than in other similar studies. However, due to the cross-sectional nature of this study and the unclear temporality of the relationship between the variables, the effect size values should be interpreted with caution. Another limitation of the study was the use of fasting glycemic index as a proxy to assess diabetes control. In this study, due to financial constraints, stronger indicators such as HbA1C were not used.

## Conclusion

A significant proportion of DM awareness and treatment can be due to the integration of diabetes in the primary health care system. The high prevalence of prediabetes and diabetes, which is affected by socioeconomic inequality and combined with low levels of glycemic control, may place a more significant burden on the health system. It is worth noting that among all people with diabetes, only 28.50% controlled their FPG level. This means, more than 70% of people were not controlled which may cause more burden of DM related complications in the future. As well as, more than 20% were unaware of their diabetes status and about 31.50% of people with diabetes do not receive any treatment. So, it is necessary to encourage people to have an annual check-up by a health physician, as well as this, periodic check-ups of blood sugar levels in people with diabetes are recommended. Therefore, it is suggested emphasizing education of patients and their physicians on trying to reach the defined goals in diabetes management.

## Data Availability

The datasets used and/or analyzed during the current study are available from the corresponding author on reasonable request.

## Abbreviations

DM: Diabetes Mellitus
SES: Socioeconomic status
WHO: World Health Organization
NCDs: Non communicable diseases
SDGs: Sustainable Development Goals
DehPCS: Dehgolan prospective cohort study
RCI: Relative concentration index
PERSIAN: Prospective epidemiological research study in Iran
BMI: Body mass index
MCA: Multiple correspondence analysis
MET: Metabolic equivalent task
SBP: Systolic blood pressure
DBP: Diastolic blood pressure
FPG: Fasting plasma glucose
CI: Confidence interval
OR: Odds ratio
